# Practical tips to fostering positive perceptions of and interest in general practice across medical training phases

**DOI:** 10.12688/mep.20921.1

**Published:** 2025-08-05

**Authors:** Faith Yong, Jordan Fox, Priya Martin, Riitta Partanen, Katharine Wallis, Matthew McGrail

**Affiliations:** 1 Rural Clinical School, The University of Queensland, Toowoomba, QLD, Australia; 2 Rural Clinical School, The University of Queensland, Rockhampton, QLD, Australia; 3 University of Southern Queensland, Toowoomba, Queensland, Australia; 4 Rural Clinical School, The University of Queensland, Hervey Bay, Queensland, Australia; 5 General Practice Clinical Unit, The University of Queensland, Herston, Queensland, Australia

**Keywords:** Workforce, general practice, career decision-making, medical specialisation, medical educators

## Abstract

**Background:**

Servicing increasing healthcare demands requires a sufficient supply of general practitioners (GPs). However, heightened by pandemic conditions, critical and chronic shortages of GPs persist globally. In light of this, new and clear strategies for promoting increased general practice/family medicine specialisation across medical education targeting emerging medical graduates are urgently needed.

**Aim and Method:**

This article aims to provide evidence-informed practical tips to foster positive perceptions of general practice and increase both interest in and uptake of general practice specialisation. These tips relate to training phases in medical school through to specialty training and are targeted at medical students, trainees, supervisors, program managers and other medical educators. They are drawn from a larger body of evidence produced by the authorship team as part of a funded project in Australia that included 25 interviews with GPs who attained their specialty fellowship between 2014-2023 and 17 key medical education stakeholders who participated in 4 facilitated workshops in late 2023.

**Conclusion:**

Through these tips, we provide a practical framework on how trainees, doctors and medical educators involved in training phases from medical school to specialty training can foster positive perceptions of and interest in general practice. These practical interventions target those from medical students, prevocational doctors and specialty registrars/residents (henceforth referred to as trainees), to their supervisors, program managers and other medical educators. These tips consider the importance of positive experiences (including language) around general practice specialisation to both encourage its uptake and to support long and successful careers in the specialty.

## Introduction

A strong general practitioner (GP) workforce is critical to the functioning of the healthcare system and health of the population. GPs are also more heavily relied upon in lower resourced settings such as rural communities whereby they take on an expanded scope of practice, as access to local specialists can be scarce ^1, 2^. In spite of the key role that GPs play, the number of medical graduates choosing to specialise in general practice/primary care/family medicine (henceforth referred to as general practice) globally falls short of community needs, with many instead choosing more specialised career pathways with a narrower scope of practice ^3– 5^. The decrease in medical graduates choosing general practice, coupled with heightened demand for GPs relating to the COVID-19 pandemic an increasing number of GPs seeking to reduce hours or retire ^6^ makes the recruitment and retention of GPs of critical and urgent importance.

Choosing a specialty is a complex decision for medical graduates, influenced by numerous job and lifestyle factors and perspectives of peers, medical educators and family members, among others ^7^. In this way, choice of specialty is developed over many years, before or during medical school and beyond. Most students will end up in a different specialty to what they are interested in when they commence medical school or even when they complete medical school ^8, 9^, particularly in some training structures (e.g., Australia, United Kingdom) where graduates commonly spend two or more years in the workforce as a junior doctor before they apply for a specialty training program. As such, it is important that trainees across all career stages have access to targeted information, mentorship, and support mechanisms to assist them in choosing an appropriate specialty. Being community-based, commonly no such comprehensive scheme exists for general practice, in contrast to other specialties whose training is primarily hospital-based. Previous work has discussed the importance of having meaningful conversations with medical students about specialty decisions ^10, 11^; this includes positive conversations about all potential options and what is likely to be the best fit for them ^10^ while also ensuring they have a realistic understanding of how competitive selection into various specialties can be ^11^. Positive role modelling can also be particularly valuable for trainees, increasing their interest in a certain specialty or simply demonstrating the type of conduct and values they may wish to exhibit as a doctor ^12^.

Given the complexity and numerous sources of information involved in specialty decision making, and the current and projected global shortage of GPs, this article aims to provide evidence-informed tips and recommendations to foster positive perceptions of general practice and increase interest in and uptake of general practice specialisation. These tips are drawn from a larger body of evidence produced by the authorship team as part of a funded project supported by the Royal Australian College of General Practitioners (RACGP) ^13^. 25 interviews were conducted between September and October 2023 with GPs who attained fellowship between 2014-2023, and 17 key stakeholders involved in medical education who participated in 4 facilitated workshops between November and December 2023. Ethics approval for the broader project was awarded 28 ^th^ August 2023 by the University of Queensland Human Research Ethics Committee (2023/HE001536). All participants provided written consent.

These tips relate to training phases from medical school to specialty training and are targeted at medical students, prevocational doctors and specialty registrars/residents (henceforth referred to as trainees), their supervisors, program managers and other medical educators. The recommendations consider the importance of positive experiences (including language) around general practice specialisation in not only encouraging the uptake of general practice but also for ensuring that GPs have long and successful careers in the specialty. Although these data are taken from an Australian context, the tips are likely to be relevant to an international audience, given that an insufficient general practice workforce is a global problem ^14^.

**Figure 1.**
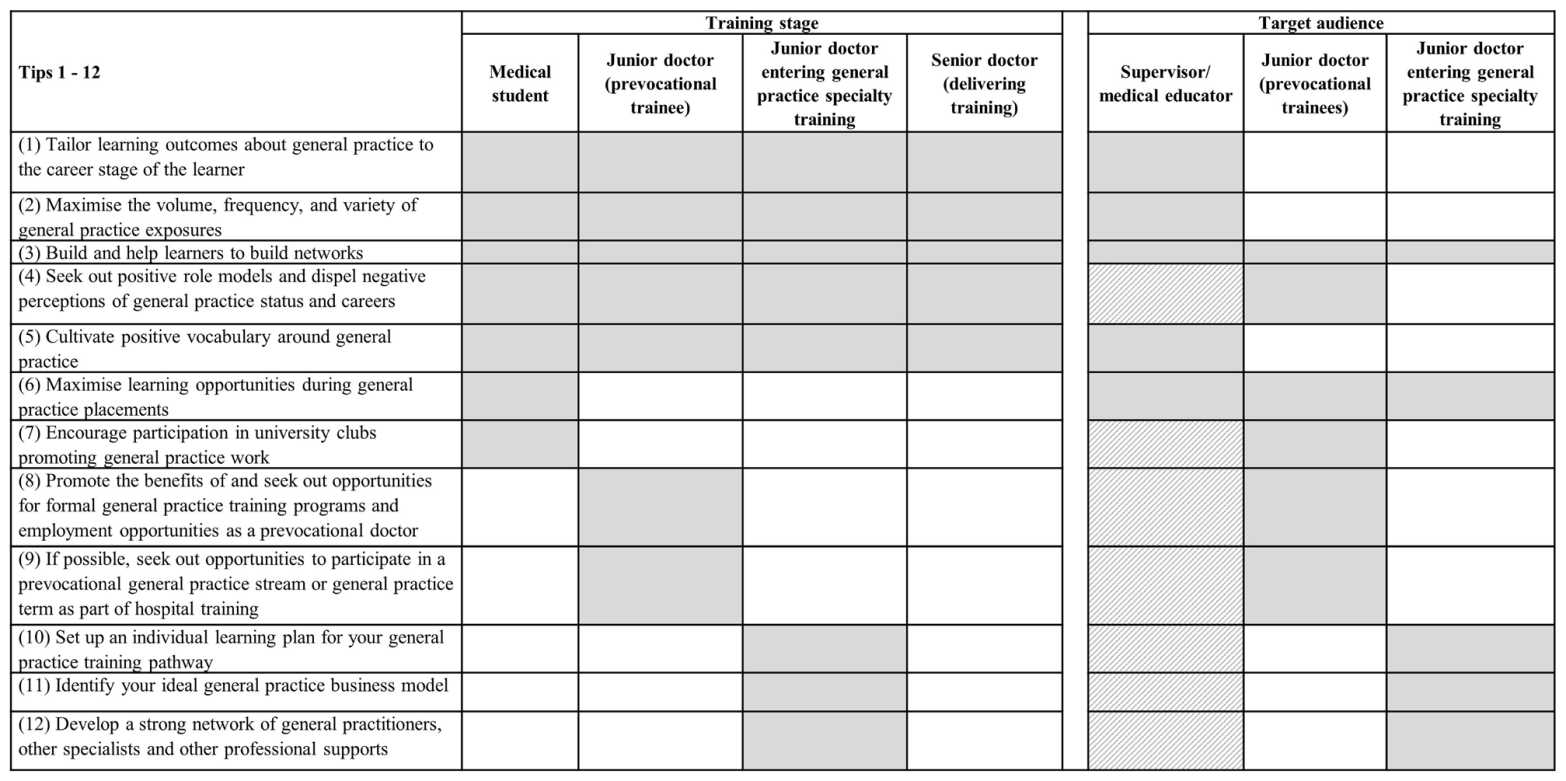
Summary of training stages and target audiences for each of the tips.

In this article, we apply ‘threshold concepts’ to inform career decision-making, defined as irreversible knowledge gain accompanied by decreased career confusion ^15^. In this regard, knowledge about specialties starts before or during medical school with learners collecting additional information across varying training phases until they decide on and solidify their choice of specialty. Figure 1 demonstrates which career stage each of the tips are most relevant and where they overlap. The figure also depicts who they are targeted at (e.g., trainees, supervisors, medical educators). The presented tips are divided into those which are relevant during all training stages (tips 1-5), medical school (tips 6-7), prevocational training (immediately prior to entering specialty training; tips 8-9), and entering general practice specialty training (tips 10-12). Tips 1, 2 and 5 are targeted at supervisors and medical educators, tips 3 and 6 are targeted at all audiences, tips 4 and 7-9 are targeted at trainees, and tips 10-12 are targeted at trainees entering general practice specialty training. The success of tips 4 and 7-12 require indirect support from involved supervisors and medical educators (hence partly shaded in Figure 1).

### Tips relevant to all training stages

***Tip 1***

**Tailor learning outcomes about General Practice to the career stage of the learner**

From being a medical student through to working as a senior doctor, medical trainees have different priorities when it comes to their learning and career progression. At different career stages they will gain varying amounts of information and have varying levels of exposure to each of the different specialties, including general practice. It is important for career advice to be relevant to career stage, to ensure it meets the needs of the learner and does not unnecessarily overwhelm them by looking too far in advance into their career ^16, 17^. Table 1, drawn from our funded project, provides a guide on the types of socialisation concerns which arise at each career stage. It can be used by all staff counselling trainees about specialty decisions and can be used by doctors supervising trainees or speaking with colleagues looking for advice.

**Table 1.** Predominant socialisation concerns by medical training stage.

Stage	Question
Medical student	What am I meant to know and do as a doctor? What do I need to do to pass my exams and assessments? How can I best learn to become a doctor?
Junior doctor (prevocational) trainee	What does it mean to be a doctor within different specialties? What kind of doctor am I? What kind of doctor do I want to be in the future? Which specialty fits with my career and life goals? Do I want to choose to be a specialist doctor? How can I enter the specialty I choose?
Specialty registrar (or residency) trainee	How can I be a doctor of the specialty I chose? How can I pass my specialty exams?
Senior doctor	As a doctor of the specialty I chose, how can or should I live my life, and conduct my career?

***Tip 2***

**Maximise the volume, frequency, and variety of general practice exposures**

Across the earlier stages of medical training in particular, medical students, interns, and junior doctors are predominantly trained in hospital environments. As a result, many report eventually choosing general practice by opting *out* of hospital specialties and working contexts, rather than opting *into* general practice. In contrast, GPs choosing to specialise because of first-hand experience of its advantages and opportunities may be a selective workforce who are more prepared and engaged ^16, 18, 19^. By medical educators creating more opportunities for exposure to general practice and by junior doctors seeking out opportunities to explore both hospital *and* non-hospital specialties early on, these career interventions can lead to much better socialisation of junior doctors in the general practice setting, resulting in gaining specific skillsets and connections whilst fully understanding what they are ‘getting themselves into’ (rather than merely opting out of hospital specialty careers) ^17, 20^. This could lead to more informed decisions being made during specialty selection, and potentially lead to an increased interest in general practice specialisation and greater retention of the GP workforce.

***Tip 3***

**Build and help learners to build networks**

A strong network of GPs and strong links between GPs and other specialists helps learners to create a sense of identity and belonging as a GP. This may include professional contacts, family members and their own GP. This network can help to develop an understanding of general practice philosophies of care, access to information regarding general practice career opportunities and sustainability, and opportunities for general practice exposure at various stages of training ^21– 23^. Becoming involved in medical education may also help to build these networks and reinforce the importance and contribution of GPs to the health system, as well as future medical education career possibilities as a GP.

***Tip 4***

**Seek out positive role models and dispel negative perceptions of GP status and careers**

Role modelling can be particularly important when making career decisions. Medical students and junior doctors are encouraged to seek out role models in general practice and hospital specialties to help them evaluate potential specialties ^12, 24– 26^. Students should take the time to evaluate the value of having a regular GP, consider what makes a great GP, start identifying suitable mentors, and define what they want from that relationship. Early in their clinical training and at the prevocational training stage, it is important to gather information to understand how GPs work and to evaluate personal experiences of the specialty as opposed to reports heard.

***Tip 5***

**Cultivate positive vocabulary around general practice**

For those supervising medical students and junior doctors, it is important to model positive behaviours in order to create positive perceptions of the various specialties, and not discourage students and junior doctors from pursuing a particular specialty ^27^. Often this starts at the top level, driven by organisational culture ^28^. As well as being a role model, each doctor involved in medical learning supervision needs to be mindful of their language surrounding general practice. Even seemingly light-hearted comments like banter can influence how individuals feel about general practice and may make trainees reluctant to explore the possibility of general practice specialisation ^29^. It may also contribute to the perception of GPs having a lower status or seen as inferior to hospital-based specialties.

### Tips relevant to medical school

***Tip 6***

**Maximise medical learning opportunities during general practice placements**

Ensuring positive experiences during general practice placements relies on the general practice supervisor providing opportunities for medical students to be proactive in maximising their learning ^30^. Medical students benefit from observing and conducting parallel consultations which allows them to take histories, complete small procedures (e.g., skin excisions and immunisations) under supervision, formulate management plans, conduct research or quality improvement projects, and ultimately become part of the practice team ^31^. Practising parallel consultations for more than 10 weeks, where possible, allows learners to understand general practice’s medical, administrative and business obligations and consequences, including fee setting decisions, continuing patient management and specialist referring decisions. These opportunities during general practice placements can form the basis of continuing connections with general practice communities, regardless of whether the student ends up in a general practice or another specialty, further contributing to positive perceptions and interest in general practice. Where possible, learning or working in general practice settings for 6–12 months can provide understanding of the rewards of longitudinal patient relationships, clinical management and ongoing care needs.

***Tip 7***

**Encourage participation in university clubs promoting general practice work**

University clubs provide a safe (non-assessed) and low stakes setting for students to make connections in the general practice space, experience general practice work and the GP community, and develop positive impressions of the general practice specialty, which is inevitably compared with their experiences in the hospital environment during medical school and as a junior doctor ^32, 33^. These clubs may also be involved with providing talks to children at primary and high schools about medical careers in various locations, which can provide exposure to new general practice communities and mentors that may be pivotal for individual career preferences. This early involvement with a general practice community leads to general practice connections, learning, and career benefits that are difficult to attain outside of and after medical school.

### Tips relevant to prevocational training

***Tip 8***

**Promote the benefits of and seek out opportunities for formal general practice training programs and employment opportunities as a prevocational doctor**

Optional general practice experiences, self-organised GP placements or locum work as a prevocational doctor provides individuals the opportunity to experience a variety of work outside of the hospital setting as an independent doctor rather than a medical student ^34, 35^. Participants in our study reported large differences in these GP experiences as a junior doctor, often gaining more insight into the specialty than as an inexperienced medical student. It also gives prevocational doctors the chance for firsthand training experiences in a variety of contexts/settings, prior to making/confirming specialty training decisions. The variety of GP skills, connections and mentors gained from such experiences may also assist in addressing concerns of GP career challenges and set them up for GP career longevity by helping them navigate unfavourable GP training obstacles.

***Tip 9***

**If possible, seek out opportunities to participate in a prevocational general practice stream or general practice term as part of hospital training**

There is considerable benefit to learning general practice philosophies (e.g., generalism, continuity of care) and gaining personal experience of general practice work as a registered independent doctor ^36, 37^. It is advantageous to do this while also having the support of a hospital position and work benefits - in Australia, general practice registrars commonly lose their hospital entitlements and support (e.g., maternity and sick leave) once they begin their community-based training. Holding a hospital position with general practice exposure allows junior doctors to explore their strengths and weaknesses in different work contexts and evaluate person-environment fit for general practice training and the lifestyles often associated with general practice or other specialties. This training structure puts junior doctors in a secure position to equitably explore general practice work and hospital-based specialties to help with their career decision-making. Even for junior doctors taking up this training structure who do not subsequently choose general practice specialisation, it is likely to further perpetuate positive narratives about the value of what GPs do and where they fit into the health system.

### Tips relevant to entering general practice specialty training

***Tip 10***

**Set up an individual learning plan for your general practice training pathway**

For junior doctors pursuing general practice specialty training, there may be considerable benefit in developing a learning plan based on supervision requirements and general practice competencies which describes how the junior doctor can best learn in the general practice environment and what still needs to be learnt from hospital placements ^38– 40^. If possible, junior doctors should identify mentors/preceptors within and outside of general practice workplaces who they can debrief with and consult when necessary.

***Tip 11***

**Identify your ideal general practice business model**

General practice organisations have different funding procedures and ways of working. Junior doctors should identify the structure which allows them to practise the type of medicine they prefer, and with the flexibility to account for personal, career, and family goals. Within general practice settings, financial viability and how it relates to patient care is the junior doctor’s personal responsibility from an earlier timepoint than other specialties. While most junior doctors no longer desire to be practice owners, they will still need to gain a degree of business prowess in order to survive outside of the hospital employment setting ^41– 43^.

***Tip 12***

**Develop a strong network of general practitioners, other specialists and other professional supports**

In hospital settings, junior doctors are encouraged to maintain connections with general practice networks, contacts and educators as it will help with career decisions ^44, 45^. General practice training is more professionally isolating than hospital training pathways, as the work and training system is different – typically general practice trainees work more autonomously and only check in with their supervisor when they really need to, as supervisors are not usually listening in on consultations or in the same room and are probably doing their own consultations. If possible, junior doctors pursuing general practice training should regularly meet with other registrars and GPs and see if their organisation will initiate weekly GP roundtables, as a means of providing necessary perspective and learnings for everyday care and the trainees’ personal wellbeing.

## Conclusions

General practice offers a rewarding and impactful medical career. Although for some, general practice can be seen as a challenging career path, there are strategies currently used to make a GP portfolio career attractive and satisfying. Increased exposure to general practice work from earlier stages in medical training and as an independent doctor can enable informed entry to the broad person-specialty fit available in the general practice specialty. These practical tips may enable better targeted interventions for individuals in medical training who are deciding upon specialty decisions and provide evidence-based initiatives for growth of general practice interest and uptake across medical training and may be protective for later general practice career retention.

## Ethics and consent

Ethics approval for the broader project was awarded 28th August 2023 by the University of Queensland Human Research Ethics Committee (2023/HE001536). All participants provided written consent.

## Data Availability

The data supporting this study, collected using individual interviews and group workshops, are stored on a secure server and only accessible by the research team as described in the data handling requirements approved by the University of Queensland’s Human Research Ethics Committee (HREC). HREC approval was contingent on data not being readily accessible beyond the named researchers. Access to data is restricted due to their risk of identifying participants (in particular, within the workshops). With the permission of the participants, access to the data may be granted upon reasonable request to the corresponding author (MM), such as specific questions related to the data set or to verify the study results. In the request, please include the reason for requesting data, plans for analysis, and contact information. Applying for access to data is initiated via the corresponding author (MM) m.mcgrail@uq.edu.au. Zenodo: Practical tips to fostering positive perceptions of and interest in general practice across medical training phases. https://doi.org/10.5281/zenodo.14997324 ^46^ This project contains the following extended data: A copy of the interview guide for semi structure interviews with GPs A copy of the workshop guide for GP training stakeholder participants Data are available under the terms of the Creative Commons Attribution 4.0 International license (CC-BY 4.0)
