# Supplementary material for: Practical tips to fostering positive perceptions of and interest in general practice across medical training phases
Source: MedEdPublish (2016). 2025 Aug 5;15:42. [Version 1] doi: 10.12688/mep.20921.1 (PMC12759286; doi:10.12688/mep.20921.1)
Supplement: Peer review history

# Reviewer report: Reviewer response for version 1

**DOI:** 10.21956/mep.22404.r44756

**Published:** 2026-01-02

**Authors:** Esther de Groot, Rianne van Diepen, Heleen Brehler

**Affiliations:** 1 UMC Utrecht, Utrecht, The Netherlands; 2 Julius Center, University of Utrecht Faculty of Medicine (Ringgold ID: 371125), Utrecht, Utrecht, Netherlands Antilles

This paper aims to provide a practical framework on how trainees, doctors and medical educators involved in training phases from medical school to specialty training can foster positive perceptions of and interest in general practice. We have read your paper with interest but, especially because we share the problem statement, on shortages of GPs and students preferring other specialisms, similar in our part of the world (Netherlands), it would be good if the paper was less a list of ideas and more founded in the literature on career decisions OR deciding that this is ‘just a tips paper’ and then leave out theory altogether.

 Our recommendations for improving the paper:

- The method section is, even for a tips paper, very brief. When recommendations are given for the whole spectrum (across training phases), one would expect that some details are given on how these respondents have an informed opinion on that.
- A bit more detail on the Australian context would be useful. Otherwise, it is hard to judge whether the tips are relevant for other countries. As an example, the GP trainee and researcher who has given feedback noticed: “outside of Australia I have no idea how common these university clubs are in Australia”. And tip 12 is different in the Dutch context, trainees have a lot of interaction with their supervisor at the General Practice where they work.
- Interesting to apply Threshold concepts. However, now, it is not very clear for people who do not know these concepts what they are and, if they do know what they are, it is not clear how applying these concepts has influenced your findings. For me, in a ‘tips’ paper, the application of theory would not be that important, you could also decide to leave it out.
- For senior doctors, we would expect that they also concern themselves with role modeling. And with lifelong learning.
- Build and help learners to build networks. We couldn’t agree more. However, the specifics, on how this is being done or could be done are missing in the section under this heading.
- Overall, acknowledging that this is a tips paper, details are missing and therefore less useful. For example the tip (under 4) to ‘gather information’ does not help.
- “each doctor involved in medical learning supervision needs to be mindful of their language surrounding general practice”. All three of us applaud this tip, but at the same time, this is not new, so perhaps some details would help, for example, putting GPs or people with knowledge about GP-practice on important educational positions within a hospital?
- Our GP trainee (and researcher) who gave feedback said “For tip 10-12 I miss how GP trainees are aided. If there is no support, shouldn’t it be a recommendation?”

**Minor details**

 Introduction

 The sentence starting with “In spite of the key role….” is very dense.

 Why are program managers not in figure 1?

If evidence from practice is presented, are all the underlying source data available to ensure full reproducibility?

Not applicable

Is the topic of the practical tips discussed accurately in the context of the current literature

Partly

Are all factual statements correct and adequately supported by citations?

No

Are the conclusions drawn balanced and justified on the basis of the presented arguments?

Partly

Are arguments sufficiently supported by evidence from the published literature and/or the authors’ practice?

Partly

Reviewer Expertise:

Area of research is in GP education.

We confirm that we have read this submission and believe that we have an appropriate level of expertise to confirm that it is of an acceptable scientific standard, however we have significant reservations, as outlined above.

# Reviewer report: Reviewer response for version 1

**DOI:** 10.21956/mep.22404.r44714

**Published:** 2025-12-30

**Authors:** Marcelo Garcia Dieguez

**Affiliations:** 1 Universidad Nacional del Sur, Bahía Blanca, Argentina

Practical-tips article drawn form a program  into 12 actions to foster positive perceptions and interest in General Practice across training phases. Even this data seems not to be published (no reference given) extended materials (interview/workshop guides) provided via Zenodo

 The main audience for this paper are  students, junior doctors, supervisors, program managers, and educators. Ethics approved

 Authors claim International relevance and pointed facts are generally sound w, many WHO report general partitioners shortage (WHO, 2018), but the but ensure consistent terminology positioning General Practice as a specialty and difference around the world, may  needs explicit boundaries and brief guidance on contextual adaptation (financing models, prevocational structures Maybe the add supporting citations where specialty status or stage definitions are discussed could help understand the boundaries.

 Paper major strengths are:

1. Clear purpose and target groups. The paper specifies who should act (trainees, supervisors, program managers) and at which stages (medical school specialty training), enabling direct uptake.
2. Actionability. Tips 11–12 illustrate practical, workplace-facing actions (identify business models; build networks/roundtables). These are specific and immediately implementable. 

Some suggestion for the authors

1. Transferability and international scope The article claims international relevance, yet evidence and implementation context are Australian A clear statement on transferability, or equivalence for other context could improve the generalizability of the tips. This could mean to specify which tips are context-free(e.g., mentorship networks) vs. context-anchored (e.g., practice business models), and provide 2–3 concrete adaptation notes for systems without extended prevocational years. I suggested to place these comments at the  end of Introduction and also in the Conclusion.

*Example sentence (Discussion)*: “Given variations in financing and prevocational pathways, Tips 9–12 may require tailoring (e.g., salaried vs. mixed-remuneration general practice). We include a Supplementary ‘adaptation guide’ with context checks and proxy metrics for non-Australian settings.”

1. Conceptual positioning  terminology on “specialty Ensure consistent framing of General Practice as a specialty and use it uniformly; when contrasting, use “non-generalist” or “hospital-based specialties” to avoid implied hierarchy. Make some relation with other denominations used around the world, if “family medicine” appears, add a parenthetical noting regional equivalence. I suggest to add a clarifying sentence early in the Introduction.  

*Example sentence (*Introduction): “In this article, we treat general practice as a medical specialty. When comparing with other fields, we use ‘non-generalist’ specialties to avoid unintended status implications.” 

1. Terminology . define “trainees” in the abstract and reuse consistently
2. Figure 1 legibility and mapping from tips→actors→stages. Current description indicates stage targeting and overlapping, but different gray color scale may need detail view to get the idea. Using cell-level labels or “+” scale could make the table more useful.

If evidence from practice is presented, are all the underlying source data available to ensure full reproducibility?

Not applicable

Is the topic of the practical tips discussed accurately in the context of the current literature

Partly

Are all factual statements correct and adequately supported by citations?

Yes

Are the conclusions drawn balanced and justified on the basis of the presented arguments?

Yes

Are arguments sufficiently supported by evidence from the published literature and/or the authors’ practice?

Yes

Reviewer Expertise:

Competency-based education, problema based learning, human resource in health, residency training

I confirm that I have read this submission and believe that I have an appropriate level of expertise to confirm that it is of an acceptable scientific standard, however I have significant reservations, as outlined above.

## References

[1] World Health Organization. Building the primary health care workforce of the 21st century. Geneva: World Health Organization; 2018 [cited 2025 Dec 15]. Available from: https://www.who.int/docs/default-source/primary-health-care-conference/workforce.pdf ​​​​​​​.

# Reviewer report: Reviewer response for version 1

**DOI:** 10.21956/mep.22404.r43206

**Published:** 2025-09-23

**Authors:** Lynn McBain McBain

**Affiliations:** 1 University of Otago, Dunedin, Otago, New Zealand

This practical tips article synthesising the literature along with recent findings from an Australian qualitative study is straightforward to read and presented in a way to enable stakeholders to select useful interventions appropriate to their role.

 The matrix presented in Figure 1 provides a visual representation of this for stakeholders .

 The tips themselves are explained succinctly and backed up with references to expand on these.

 The article is useful reading for all involved in training for the medical workforce.

 Some specific comments which may assist clarity:

**Abstract:**

 There is repetition in the section aim and method and the section conclusion.

 I think it could be rewritten so as not to be so repetitive – for example – define the term “trainees“ in the aim part and then use that term in the conclusion when emphasising the range of those  targeted by the intervention.

**Introduction:**

 Please take care in ensuring that GPs are referred to as specialists throughout.

 Sentence 1: …expanded scope of practice, as access to local specialists can be

 scarce…

 Is there another word that can describe the local non-GP doctors perhaps consultants – remembering that GPs are specialists in their own right (as the authors state in the very next sentence).

 And then again Sentence 2 refers to specialised career pathways implying these are different to general practice pathways.   Perhaps reword to… non-generalist career pathways with a narrower scope of practice ….

 In **Figure 1** it is very difficult to see the different in shading referred to in the text for tips  4 and 7-12.

 In the explanation of each tip there is cited literature to inform the discussion.

 The tips themselves with accompanying Table and Figure are very useful.

 This is a helpful overview and should be part of the toolkit for all medical schools particularly general practice departments / sections to champion.

If evidence from practice is presented, are all the underlying source data available to ensure full reproducibility?

Not applicable

Is the topic of the practical tips discussed accurately in the context of the current literature

Yes

Are all factual statements correct and adequately supported by citations?

Yes

Are the conclusions drawn balanced and justified on the basis of the presented arguments?

Yes

Are arguments sufficiently supported by evidence from the published literature and/or the authors’ practice?

Yes

Reviewer Expertise:

Primary health care

I confirm that I have read this submission and believe that I have an appropriate level of expertise to confirm that it is of an acceptable scientific standard.
